# Cause and prevention of demyelination in a model multiple sclerosis lesion

**DOI:** 10.1002/ana.24607

**Published:** 2016-02-22

**Authors:** Roshni A. Desai, Andrew L. Davies, Mohamed Tachrount, Marianne Kasti, Frida Laulund, Xavier Golay, Kenneth J. Smith

**Affiliations:** ^1^ Department of Neuroinflammation and Queen Square Multiple Sclerosis CentreUCL Institute of NeurologyLondonUnited Kingdom; ^2^ Department of Brain Repair and RehabilitationUCL Institute of NeurologyLondonUnited Kingdom

## Abstract

**Objective:**

Demyelination is a cardinal feature of multiple sclerosis, but it remains unclear why new lesions form, and whether they can be prevented. Neuropathological evidence suggests that demyelination can occur in the relative absence of lymphocytes, and with distinctive characteristics suggestive of a tissue energy deficit. The objective was to examine an experimental model of the early multiple sclerosis lesion and identify pathogenic mechanisms and opportunities for therapy.

**Methods:**

Demyelinating lesions were induced in the rat spinal dorsal column by microinjection of lipopolysaccharide, and examined immunohistochemically at different stages of development. The efficacy of treatment with inspired oxygen for 2 days following lesion induction was evaluated.

**Results:**

Demyelinating lesions were not centered on the injection site, but rather formed 1 week later at the white–gray matter border, preferentially including the ventral dorsal column watershed. Lesion formation was preceded by a transient early period of hypoxia and increased production of superoxide and nitric oxide. Oligodendrocyte numbers decreased at the site shortly afterward, prior to demyelination. Lesions formed at a site of inherent susceptibility to hypoxia, as revealed by exposure of naive animals to a hypoxic environment. Notably, raising the inspired oxygen (80%, normobaric) during the hypoxic period significantly reduced or prevented the demyelination.

**Interpretation:**

Demyelination characteristic of at least some early multiple sclerosis lesions can arise at a vascular watershed following activation of innate immune mechanisms that provoke hypoxia, and superoxide and nitric oxide formation, all of which can compromise cellular energy sufficiency. Demyelination can be reduced or eliminated by increasing inspired oxygen to alleviate the transient hypoxia. Ann Neurol 2016;79:591–604

The events responsible for the formation of new inflammatory demyelinating lesions in multiple sclerosis (MS) remain unknown.^1^ Many investigators favor an autoimmune mechanism, but rather than developing like the autoimmune lesions of experimental autoimmune encephalomyelitis, the most commonly used model of MS, newly forming lesions in MS show a relative paucity of T cells,^1^, ^2^, ^3^ which are reported to arrive later in lesion development.^2^, ^3^ Early lesions have been described by different investigators as prephagocytic,^1^ primary,^3^ or pattern III,^4^ and the associated demyelination has distinctive characteristics (see below).

Pathological studies have implicated reactive oxygen and nitrogen species^5^ in lesion formation, and have suggested that such agents may impair mitochondrial metabolism, resulting in a tissue energy deficiency,^6^ a mechanism later termed “virtual hypoxia.”^7^ The distinctive early demyelination, characterized partly by preferential loss of myelin‐associated glycoprotein,^4^ has been described as “hypoxia‐like” due to factors such as the expression of hypoxia‐related antigens,^8^, ^9^ including the prominent nuclear expression of hypoxia‐inducible factor‐1α (HIF‐1α).^10^ Oligodendrocytes are notably vulnerable to an energy deficit, as they not only maintain numerous internodes of myelin, which is a substantial metabolic load in itself, but also provide metabolic support for axons.^11^ Systemic exposure to carbon monoxide impairs oxygen delivery and mitochondrial function throughout the body, but it selectively causes cerebral demyelination.^12^ Oligodendrocytes may also suffer, even at the earliest stages of lesion formation, from at least some of the mitochondrial impairments that have been described in established MS lesions.^13^

Lesions have a predilection to form in the periventricular and juxtacortical regions,^14^ the spinal white matter tracts,^15^ and the optic nerves.^16^ In a large study of 1,594 plaques, Brownell and Hughes^17^ noted that periventricular lesions “have the peculiarity that they are situated on the boundary zones between major cerebral arteries which have penetrated in this periventricular region to their further point of supply,” namely the watershed areas between the anterior, middle, and posterior cerebral arteries, and this observation has recently been confirmed.^18^ Presciently, from the standpoint of the current observations, Brownell and Hughes commented, “It may be of aetiological significance that the sites where plaques were commonly formed are areas where relative vascular insufficiency can be postulated.”^17^ Furthermore, vascular injection studies^19^ have highlighted that sites of lesion formation tend to contain few vessels, with those present derived from 2 independent major arteries that have reached their furthest point. Such watershed regions have prolonged arterial transit times,^20^ and this may render them vulnerable to impaired perfusion. In agreement, a recent magnetic resonance imaging (MRI) examination of 1,249 cases of MS^21^ observed that lesions tended to accumulate in regions with relatively lower perfusion than normal‐appearing white matter, and furthermore the MS brain may exhibit poor perfusion.^22^, ^23^, ^24^ Attention has also been drawn to the common occurrence of lesions at the junction between the gray matter of the cortex and the underlying white matter.^17^

In addition to a relationship with watershed regions, it has long been apparent that new lesions tend to form around veins,^25^ which has encouraged the suspicion that deleterious factors emanate from veins to disturb the surrounding tissue (see Prineas and Parratt^1^, but the particular factors have remained elusive.

Here, we employ an in vivo model of the early MS lesion^26^, ^27^, ^28^ to explore the mechanisms involved in the demyelination, and whether it can be prevented.

## Materials and Methods

### Lipopolysaccharide Lesion Induction

In brief, a quarter laminectomy was performed aseptically between the T12 and T13 vertebrae in adult male Sprague Dawley rats (312g ± 31.9, mean ± standard deviation), under deep isoflurane anesthesia (2% in oxygen), as described previously.^26^ A glass micropipette was used to microinject lipopolysaccharide (LPS; 0.5 μl of 100ng/µl in saline; *Salmonella enterica* abortus equi; Sigma‐Aldrich, St Louis, MO) into the right dorsal column at depths of 0.7 and 0.4mm (n = 3 rats per time point); control animals received injections of saline alone (n = 2 per time point). The injection site was marked with charcoal on the dura for subsequent histological localization.

### Exposure of Naive Animals to Hypoxia

Female Dark Agouti rats (163.7g ± 7.8), were exposed to normobaric hypoxia by substituting oxygen with nitrogen using a ProOx 110 controller (Biospherix, Salem, NY) in a purpose‐designed chamber (Biospherix). The hypoxia was gradually introduced by decreasing the oxygen from 21 to 10% over 20 minutes, prior to continuous exposure to 10% oxygen for 6 hours (n = 3) or 24 hours (n = 6). Animals housed in the same chamber, in room air (21% oxygen; n = 6), served as controls.

### In Vivo Detection of Hypoxia and Superoxide Production

The intravenous probe pimonidazole (HPI, Burlington, MA) was employed to detect tissue hypoxia in both studies, as previously described.^29^ The intravenous probe dihydroethidium (DHE; Sigma‐Aldrich) was used to indicate superoxide production in the LPS dorsal column study. DHE is thought to react with superoxide to produce 2‐hydroxyethidium, which intercalates with DNA, resulting in a red fluorescence. Pimonidazole (180mg/kg [naive hypoxia study] or 60mg/kg [LPS dorsal column study]) and DHE (1 μg/ml in dimethylsulfoxide) were administered into different saphenous veins under brief anesthesia (2% isoflurane) with recovery, 4 hours prior to perfusion, as previously described.^29^

### Normobaric Oxygen Therapy

To examine the consequence of increasing the concentration of inspired oxygen on LPS‐induced demyelination, animals were randomized into treatment groups exposed to either room air (n = 8) or normobaric hyperoxia (80% oxygen, n = 11) for 2 days following the injection of LPS into the dorsal columns. Animals were housed in a purpose‐built chamber (BioSpherix) for the duration of the treatment, and temperature, oxygen concentration, and carbon dioxide concentration were monitored and controlled throughout. Following treatment, animals were returned to their home cages and maintained at room air until perfusion, 12 days later.

All the protocols involving animals were approved by the institutional ethics committee, licensed under the UK Animals (Scientific Procedures) Act of 1986, and conducted according to the ARRIVE guidelines. Animals were provided with food and water ad libitum throughout.

### Perfusion and Tissue Collection

All animals were transcardially perfused with rinse solution (0.9% NaCl, 2,000U/l heparin, 0.025% lidocaine, 0.02% 4‐[2‐hydroxyethyl]‐1‐piperazineethanesulfonic acid [pH 7.4]) followed by paraformaldehyde (4% in 0.15M phosphate buffer) under deep anesthesia (3% isoflurane) after 6 or 24 hours of exposure to 10% oxygen (naive hypoxia study), and at 12 hours or 1, 2, 3, 7, or 14 days after LPS injection (LPS lesion study). To visualize the spinal cord vasculature, animals exposed to 6 hours of 10% oxygen were additionally perfused with the fluorescent carbocyanine lipophilic dye, DiI (Molecular Probes, Eugene, OR) after perfusion with rinse solution but prior to perfusion–fixation.

### MRI Protocol

Fixed spinal cords were washed in phosphate‐buffered saline (PBS) prior to arrangement in a custom‐built sample holder and immersed in Fomblin perfluoropolyether (LC 08; Solvay Solexis, Milan, Italy) to avoid susceptibility artifacts. The holder was positioned in a horizontal bore 9.4T preclinical MRI scanner (Agilent, Santa Clara, CA) equipped with a 33mm‐diameter radiofrequency birdcage volume coil (Rapid Biomedical, Rimpar, Germany). Images with a spatial resolution of 50 × 50 × 300 µm were acquired using a volumetric gradient echo sequence with the following parameters: echo time = 20 milliseconds, repetition time = 100 milliseconds, flip angle = 35 °, field of view = 28 × 14 × 38.4mm, matrix size = 560 × 280 × 128, number of averages = 12, and a total acquisition time = 12.5 hours.

### Tissue Processing and Histology

All tissue was postfixed overnight in either 4% paraformaldehyde for cryosections, followed by cryoprotection in 30% sucrose in PBS, or 4% glutaraldehyde in 0.15M phosphate buffer for resin sections, and subsequently processed for examination in frozen or semithin resin sections using standard techniques and the range of antibodies described in the Supplementary Table, as previously described.^26^, ^29^

### Microscopy and Quantification

#### Light Microscopy

Tissue labeled using the peroxidase detection system was viewed using an Axiophot light microscope (Carl Zeiss, Oberkochen, Germany) and photographed with a Nikon D300 camera (Nikon Instruments, Melville, NY).

#### Confocal Laser Microscopy

Fluorescent images were obtained using a Zeiss LSM5 Pascal confocal microscope, with ×2.5 and ×40 objectives.

#### Quantification

All analysis and quantification was performed blind using ImageJ (NIH, Bethesda, MD). Cells positively labeled with adenomatous polyposis coli (APC), HIF‐1α, and inducible nitric oxide synthase (iNOS) were counted in the spinal dorsal column at the site of injection and expressed as cell density. Pimonidazole labeling was quantified as described previously.^29^ Lesion size in magnetic resonance images was determined using a threshold intermediate to nonlesion gray and white matter contrast and manually delineated for each scan frame. Regions of myelin loss in Luxol fast blue (LFB)‐stained sections were manually circumscribed and expressed as a percentage of dorsal column area.

### Statistical Analysis

All data were tested for normality using either the Kolmogorov–Smirnov or the Shapiro–Wilk test, and for homogeneity of variances using the Levene test. Linear regression analysis was used to compare differences between groups for the different immunohistochemical markers, and when significant, further compared using independent *t* tests between groups at each individual time point. Independent *t* tests were also used to compare lesion length, maximum cross‐sectional area, and volume between oxygen and room air treatment groups. A Pearson correlation coefficient was used to assess reliability of MRI measures of lesion size in comparison with LFB staining. Probability values of <0.05 were considered statistically significant. All statistical analyses employed SPSS (IBM, Armonk, NY).

### Three‐Dimensional Reconstruction

To study the relationship between the distribution of pimonidazole labeling and the vasculature, 3‐dimensional (3D) reconstruction was performed on serial sections that were double‐labeled with antipimonidazole and anti–RECA‐1 antibodies using the Reconstruct editor (Boston University, Boston, MA).

## Results

### Demyelination

**Figure 1:**
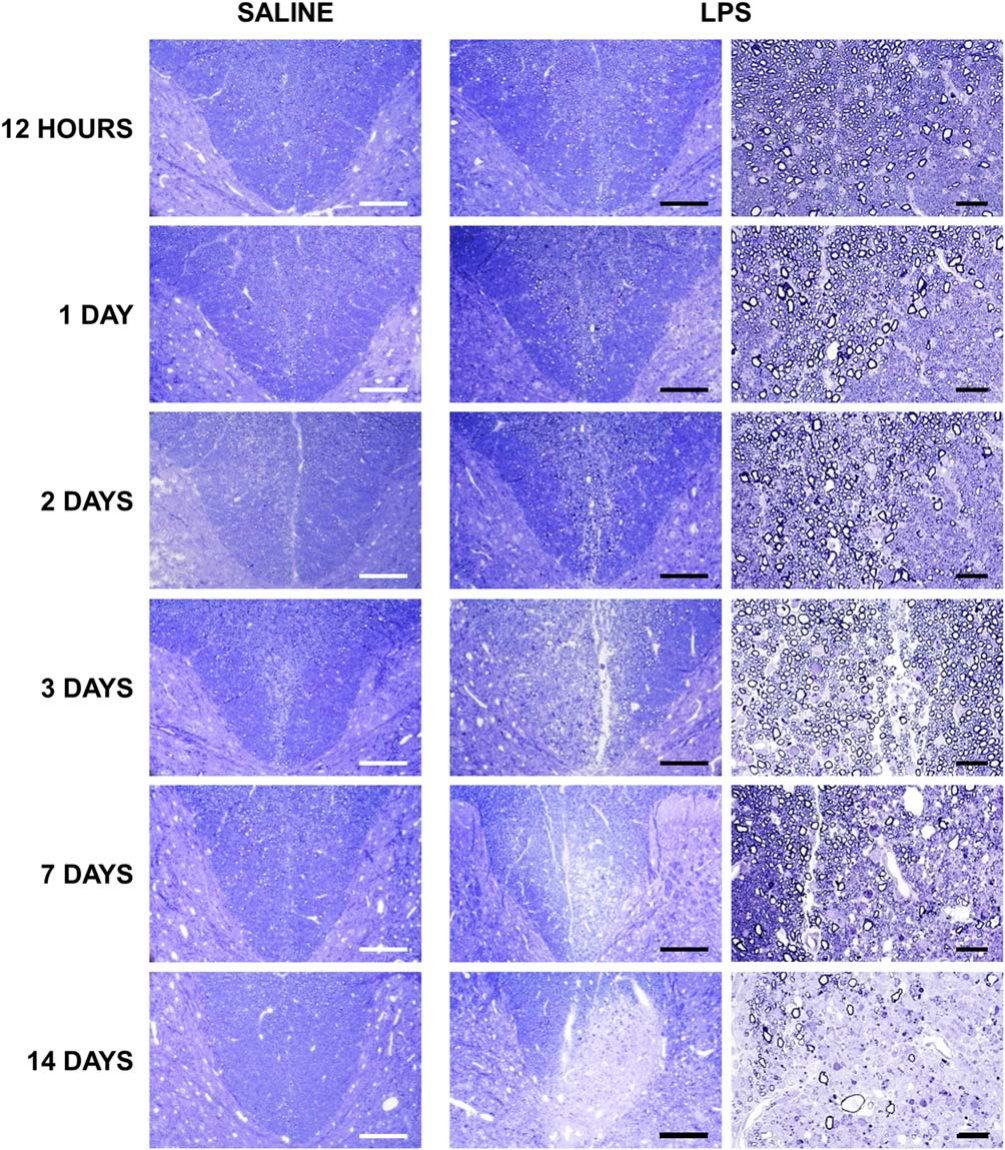
Time course of lesion formation following intraspinal lipopolysaccharide (LPS) injection. Light micrographs of transverse, semithin resin sections of spinal cords show the base of the dorsal columns at the level of the injection of saline or LPS. Tissue integrity is maintained following the injection of saline, with no apparent pathology. In the acute LPS lesion (12 hours to 2 days postinjection), the tissue appears grossly unaffected, although some inflammatory cells are apparent. At 3 days postinjection, the tissue is edematous, with some debris‐containing macrophages present in the dorsal columns of LPS‐injected animals. At 7 days postinjection, the lesion remains edematous, with the appearance of some demyelinated axons. By 14 days, the lesion contains many demyelinated axons, several debris‐filled macrophages, and a few axons undergoing degeneration. Scale bars = 200 µm (first and second columns) and 100 µm (last column). All micrographs are representative.

The unilateral injection of LPS into the dorsal column white matter of the rat spinal cord induced a focal primary demyelinating lesion, as described previously (Fig 1).^26^, ^27^ At early time points after injection (12 hours, 1 day, and 2 days), the tissue appeared grossly normal when examined in resin sections, although closer inspection revealed evidence of inflammation within the dorsal white matter. By the third day after injection there was obvious edema present, particularly at the base of the dorsal columns, but the myelin appeared intact. The edema persisted 7 days after injection, with clear evidence at this time of primary demyelination, which consistently involved the tissue on either side of the white matter–gray matter boundary ipsilateral to the injection, sometimes extending to involve the most ventral dorsal columns, bilaterally. Debris‐filled macrophages were observed within the lesion. By 14 days postinjection, lesions were comprised of many demyelinated axons and debris‐filled macrophages, but no evidence of edema was observed. The lesion in this study closely resembled that described in more detail previously.^26^, ^27^, ^28^, ^30^

**Figure 2:**
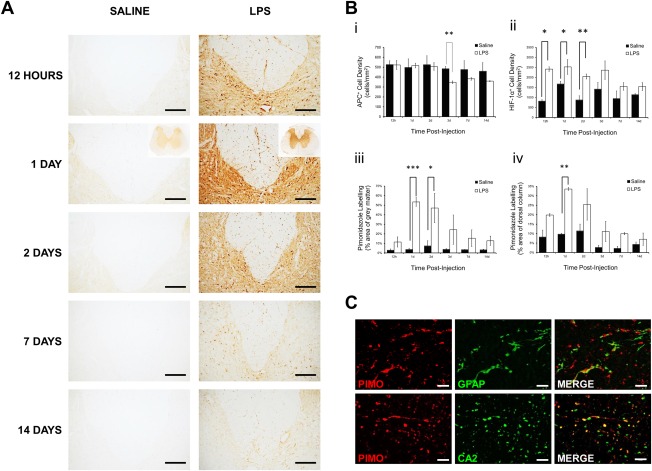
Immunohistochemical examination and quantification of labeling for bound pimonidazole adducts, adenomatous polyposis coli (APC), and hypoxia‐inducible factor‐1α (HIF‐1α), following the injection of saline or lipopolysaccharide (LPS) into the dorsal columns. (A) Representative images showing the base of the dorsal columns and adjacent gray matter at the level of injection after 12 hours, and 1, 2, 7, and 14 days. Labeling for pimonidazole is increased bilaterally in both the gray and white matter of animals injected with LPS, compared with saline‐injected controls, particularly at 1 day following injection, with insets showing the spinal cord at lower magnification. The gray matter labels for bound pimonidazole following LPS injection, and individual cells are labeled throughout the white matter, but particularly within the dorsal and dorsolateral columns. The labeling in LPS‐injected animals is particularly intense in the gray matter immediately adjacent to the dorsal columns. (Bi) Graphical representation of APC‐positive oligodendrocyte density in the entire dorsal columns at different time points after injection (saline n = 2, LPS n = 3, per time point). Oligodendrocyte loss was apparent 3 days after LPS injection. (B) Graphical representation of the density of HIF‐1α–positive cells in the entire dorsal column at different time points after injection (saline n = 2, LPS n = 3, per time point; Bii). Graphical representation is shown of the intensity of pimonidazole labeling of the gray matter (Biii) and dorsal column (Biv) at different time points after injection (saline n = 2, LPS n = 3, per time point; mean ± standard error of the mean [SEM]). Statistical significance was determined by independent *t* test, comparing saline‐injected and LPS‐injected animals at each time point (*p < 0.05, **p < 0.01, ***p < 0.001). (C) Double label immunofluorescence with antibodies against pimonidazole (PIMO; red), and glial fibrillary acidic protein (GFAP; astrocytes) or carbonic anhydrase 2 (CA2; oligodendrocytes; green), showing that a subset of astrocytes and oligodendrocytes in the dorsal column white matter label positively for pimonidazole at 1 day following LPS injection. Scale bars = 200 µm (A), 500 µm (A *insets*), 20 µm (C).

Oligodendrocyte number was assessed using an antibody directed against the marker tumor suppressor APC. The number of oligodendrocytes in the dorsal columns of animals injected with LPS was comparable to saline‐injected controls during the first 2 days, but was significantly decreased at 3 days (*p* < 0.01; Fig 2Bi); no change in oligodendrocyte number was observed in saline‐injected controls.

### Pimonidazole

Immunoreactivity for pimonidazole was prominent throughout the spinal cord at the level of LPS injection from 12 hours to 2 days after lesion induction, several days prior to the onset of demyelination (see Fig 2). Labeling was most obvious in the gray matter, especially adjacent to the injection, but intense punctate labeling of glial cells was also present throughout the white matter, particularly within the dorsal columns; pimonidazole does not label myelin, so the white matter misleadingly appears at first glance to be less intensely labeled than the gray matter. Quantification revealed that labeling in the gray matter was significantly elevated in animals injected with LPS at 1 day (*p* < 0.001) and 2 days (*p* = 0.014) postinjection, compared with saline‐injected controls. Labeling for pimonidazole in the dorsal column white matter was significantly greater (*p* < 0.005) than controls at 1 day after LPS injection, and double‐label immunofluorescence revealed colabeling of pimonidazole with carbonic anhydrase 2, a marker of oligodendrocytes, and glial fibrillary acidic protein, a marker of astrocytes. No such labeling was present in saline‐injected controls.

### HIF‐1α

**Figure 3:**
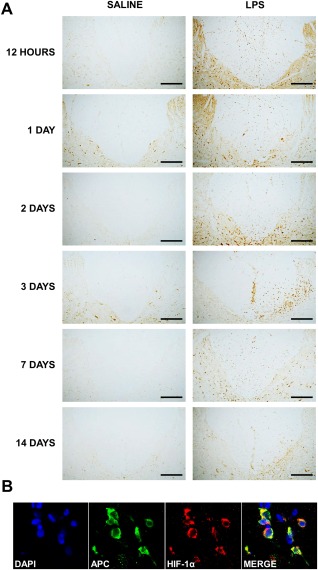
Immunohistochemical examination of the expression of hypoxia‐inducible factor‐1α (HIF‐1α) following the injection of saline or lipopolysaccharide (LPS) into the dorsal columns. (A) Representative micrographs of the dorsal columns and the adjacent gray matter at the level of injection of saline or LPS, labeled with an antibody against HIF‐1α. Basal HIF‐1α immunoreactivity is evident in the dorsal white matter of saline‐ and LPS‐injected animals at all time points, but is increased following LPS injection. HIF‐1α–positive cells are scattered throughout the dorsal columns at 12 hours and 1 day following LPS injection, but become more focused at the base of the dorsal columns ipsilateral to the injection after 2 days. (B) Double label immunofluorescence with antibodies against adenomatous polyposis coli (APC) (oligodendrocytes; green) and HIF‐1α (red), counterstained with 4,6‐diamidino‐2‐phenylindole (DAPI) for nuclei (blue) at 24 hours after LPS injection. Scale bars = 200 µm (A) and 20 µm (B).

The pimonidazole findings were corroborated using immunohistochemistry for the endogenous marker HIF‐1α. HIF‐1α–positive cell counts were increased in the dorsal columns of LPS‐injected animals (Figs 3A, 2Bii) at 12 hours (*p* = 0.011), 1 day (*p* = 0.029), and 2 days (*p* = 0.002) postinjection, compared with saline‐injected controls. HIF‐1α labeling was observed in the cytoplasm and nuclei of cells throughout the dorsal columns and adjacent gray matter 12 hours to 2 days after LPS injection; these were identified as oligodendrocytes based on double label immunofluorescence (see Fig 3B). By 3 days after injection, HIF‐1α labeling was restricted to the ipsilateral white matter–gray matter border.

### Superoxide

**Figure 4:**
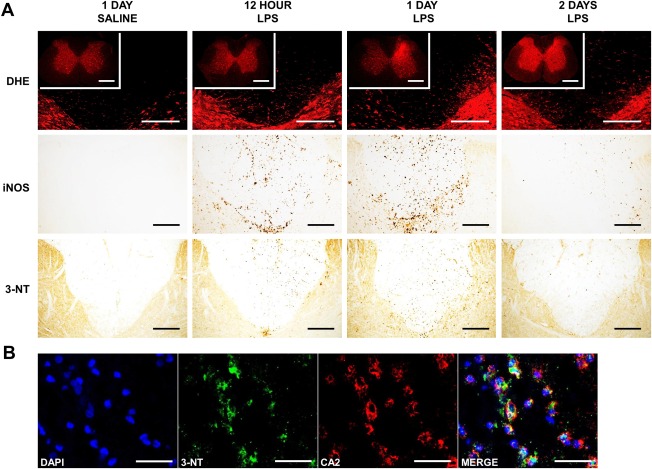
Oxidative and nitrosative stress in the lipopolysaccharide (LPS) dorsal column lesion. (A, Top) Representative confocal micrographs showing the base of the dorsal columns and adjacent gray matter at the level of the injection of saline and LPS, 12 hours, and 1 and 2 days postinjection, examined for superoxide‐induced fluorescence (DHE), with insets showing the spinal cord at lower magnification. Following the injection of LPS, there is an increase in superoxide‐induced fluorescence in both the white and gray matter at each of the time points examined. The increase is particularly intense ipsilateral to the site of injection at 1 day in the white matter and adjacent gray matter, and more generalized at 2 days. (A, Middle) Immunoreactivity for inducible nitric oxide synthase (iNOS) is absent in control tissue, but prominent in the dorsal columns, particularly in the adjacent gray matter at 12 and 24 hours following the injection of LPS. Cells positive for iNOS also cluster at the base of the dorsal columns and in the immediately adjacent gray matter. Labeling for iNOS is decreased by 2 days after injection. (A, Bottom) Representative micrographs of spinal cord sections showing the dorsal columns at the level of the injection of saline or LPS, labeled with an antibody against 3‐nitrotyrosine (3‐NT). In the acute lesion (12 hours to 2 days), immunoreactivity for 3‐NT is evident in the dorsal columns of LPS‐injected animals, but is absent in saline‐injected controls. Positive cells can be seen dispersed throughout the dorsal white matter, but the labeling is most intense ipsilateral to the site of injection, at the base of the dorsal columns, and in the adjacent gray matter. (B) Double label immunofluorescence with antibodies against 3‐NT (green) and carbonic anhydrase 2 (CA2; oligodendrocytes; red), counterstained with 4,6‐diamidino‐2‐phenylindole (DAPI; nuclei; blue) at 24 hours after LPS injection. Scale bars = 200 µm (A; 500 µm in DHE *insets*) and 20 µm (B).

DHE fluorescence was observed at a basal level throughout the spinal cords of all saline‐injected animals, but was increased in the spinal cords of animals injected with LPS by 1 day post‐injection, and focused on either side of the white matter–gray matter border adjacent to the injection site and involving the base of the dorsal columns (Fig 4A). By 2 days, the observed superoxide‐induced fluorescence was still greater than in saline‐injected controls, but more uniformly distributed throughout the gray and white matter.

### Nitric Oxide

Counts of iNOS‐positive cells were significantly greater in the dorsal columns of animals injected with LPS at 12 hours (*p* < 0.001), 1 day (*p* < 0.001), and 2 days (*p* = 0.011) following injection, compared with saline‐injected animals, with the greatest density observed at 1 day (see Fig 4A). Cells positive for iNOS were dispersed diffusely throughout the dorsal columns of LPS‐injected animals. Positive cells were also found in the adjacent gray matter, with a dense cluster localized to the white matter–gray matter border at the base of the dorsal columns and ipsilateral to the injection. No such labeling was present in saline‐injected controls, or in LPS‐injected animals by the second day after injection.

### Nitrotyrosine

The formation of peroxynitrite was disclosed via the immunohistochemical detection of nitrotyrosine residues. Immunoreactivity in the dorsal columns of LPS‐injected animals conformed to the spatiotemporal pattern of iNOS and superoxide labeling. Labeling was present 12 hours after LPS injection, but most prominent at 1 day, before decreasing by 2 days (see Fig 4), and was notably evident in oligodendrocytes (see Fig 4B). No labeling for nitrotyrosine was evident in saline‐injected controls, or in LPS‐injected animals by the third day after injection.

### Spatial and Cellular Vulnerabilities to Hypoxia

**Figure 5:**
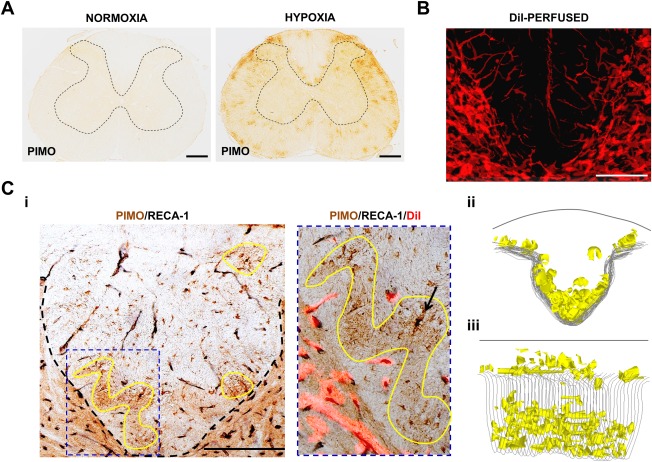
Regional vulnerability of the naive spinal cord to hypoxia. (A) Spinal cord sections from naive rats exposed to 21% (room air) or 10% oxygen in conjunction with systemically administered pimonidazole (PIMO). In room air there is no labeling for tissue hypoxia, but there is prominent labeling for bound pimonidazole when breathing 10% oxygen. The labeling primarily occurs in patches in the spinal white matter, particularly around the base and dorsolateral edges of the dorsal columns. The outline of the gray matter is indicated by dashed lines. (B) Superimposed confocal micrographs of adjacent spinal cord sections from an animal perfused with DiI, showing the base of the dorsal columns and the adjacent gray matter. The rich vascular density in the gray matter and the relative paucity of vessels in the dorsal column white matter are evident, particularly at the base of the dorsal columns. (Ci) Transverse section of spinal cord from a rat exposed to 10% oxygen, and immunolabeled with pimonidazole (brown) and RECA‐1 (black; endothelial cell marker), showing areas of intense pimonidazole labeling at the base of the dorsal columns, some of which are outlined in yellow. The image to the right shows one of the outlined regions at higher magnification, superimposed on a fluorescent image of the same section showing perfused vessels labeled with DiI (red fluorescence). The endothelial labeling coincides with the DiI labeling, indicating both the presence of endothelial cells and blood perfusion. The outlined area of intense pimonidazole labeling is devoid of blood vessels (as revealed by the absence of RECA‐1 and DiI labeling). A glial cell *(arrow)* labeled intensely with pimonidazole (brown) is distinguished from a blood vessel (black) by the absence of DiI labeling. (Cii, Ciii) Three‐dimensional reconstruction of the dorsal columns from an animal exposed to 10% oxygen. The reconstruction shows the location of areas of intense pimonidazole labeling (yellow) obtained from a series of adjacent transverse sections taken over a 1mm length of spinal cord, as viewed from the head to the tail (Cii), or obliquely (Ciii). Scale bars = 500 µm (A) and 200 μm (B, C). Micrographs are representative.

Exposure to 10% oxygen was used to disclose anatomical and cellular vulnerabilities to hypoxia in the naive rat spinal cord. Accordingly, labeling for pimonidazole was negative in the spinal cord of animals breathing room air (n = 3), but was prominent in all animals breathing 10% oxygen for 6 hours (Fig 5A) or 24 hours (data not shown; n = 3). The white matter was selectively affected, with labeling preferentially located along the white matter–gray matter border at the base of the dorsal columns, in the white matter adjacent to the dorsal root entry zone, and in subpial areas throughout the cord. Visualization of the spinal vascular network with DiI revealed a relative scarcity of vessels in these same white matter locations (see Fig 5B). Sections used for DiI vessel location were then subjected to double label immunohistochemistry for pimonidazole and RECA‐1, revealing that regions labeled with pimonidazole were typically located between blood vessels (see Fig 5Ci). 3D reconstruction of these areas revealed the vascular vulnerability of the base of the dorsal columns and the white matter–gray matter border to hypoxia.

**Figure 6:**
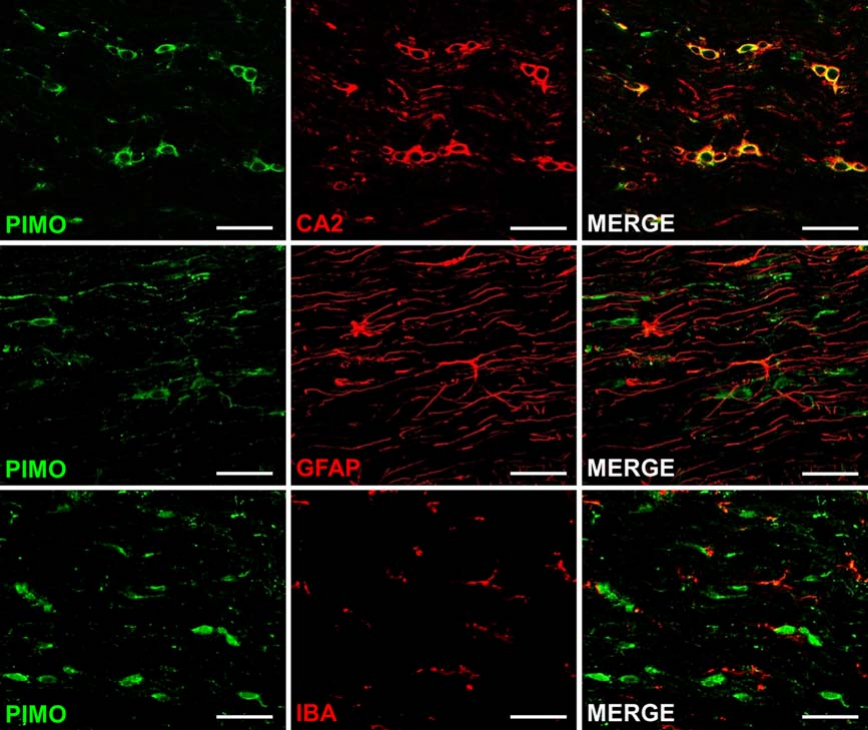
Cellular vulnerabilities to hypoxia in the naive spinal cord. Confocal fluorescence images show spinal cord sections from animals exposed to 10% oxygen for 24 hours, double‐labeled for pimonidazole (PIMO; green), and carbonic anhydrase 2 (CA2; oligodendrocytes; red), glial fibrillary acidic protein (GFAP; astrocytes; red), or ionized calcium‐binding adapter molecule 1 (IBA; microglia; red). Colocalization indicates that oligodendrocytes, but neither astrocytes nor microglia, selectively label for pimonidazole during exposure to 10% oxygen. Scale bars = 100 μm. All micrographs are representative.

Exposure to 10% oxygen for 6 (n = 3; data not shown) or 24 hours (n = 6; Fig 6) resulted in clearly defined cell‐specific pimonidazole labeling in the spinal cord white matter. Double‐label immunofluorescence in longitudinal sections revealed arrays of glial cells with selective oligodendrocyte labeling with pimonidazole; astrocytes and microglia were not labeled (see Fig 6).

### Oxygen Therapy and Demyelination

**Figure 7:**
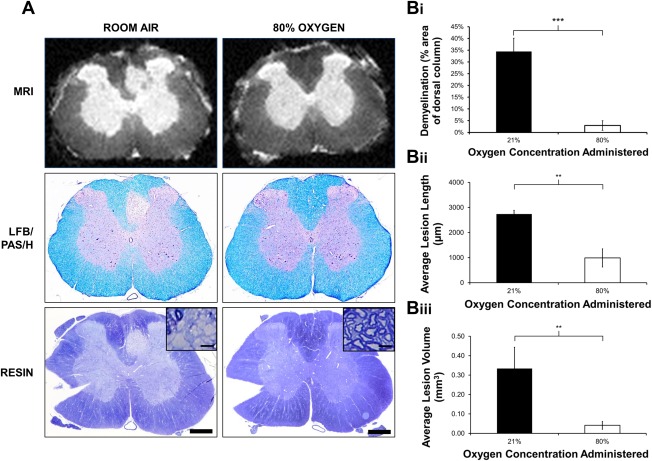
Oxygen therapy and demyelination. (A) Representative ex vivo magnetic resonance images of Luxol fast blue (LFB)/periodic acid–Schiff (PAS)/hematoxylin (H)‐stained cryosections and resin‐embedded sections of the lesion epicenter of LPS‐injected animals exposed to either room air or 80% oxygen, showing that treatment with 80% oxygen decreases the size of demyelinating lesions. Many demyelinated axons are evident following treatment with room air *(inset)*, whereas rims of myelin appear to be preserved around axons in animals treated with oxygen *(inset)*. (Bi) Graphical representation of the average size of demyelinated lesions (as a percentage of the entire dorsal column area) in LFB/PAS/H‐stained sections showing significantly smaller demyelinated lesions in animals treated with 80% oxygen (n = 11) compared with those treated with room air (n = 8; mean ± SEM). (Bii, Biii) Quantification of magnetic resonance images revealed that lesion length (Bii) and lesion volume (Biii) are also significantly reduced following treatment with 80% oxygen, compared to room air controls (mean ± SEM). Statistical significance was determined by independent *t* test (**p < 0.01, ***p < 0.001). Scale bars = 500 µm (100 µm in *insets*). Note: notches in resin sections were used to confirm the side of injection during processing. MRI = magnetic resonance imaging.

Characteristic primary demyelinating lesions were observed at the gray–white matter border in control animals maintained in room air (n = 8) for 14 days following intraspinal LPS injection. In contrast, the area of demyelination was dramatically and significantly (*p* < 0.001) reduced, or even absent, in rats treated with 80% oxygen during the first 2 days after LPS injection (n = 11), as determined by the loss of LFB staining (Fig 7). The histochemical results were confirmed by analysis of resin sections, and magnetic resonance images, the latter of which revealed that both the length and 3D volumes of lesions in animals exposed to 80% oxygen were significantly reduced (length, *p* = 0.003; volume, *p* = 0.010), compared with room air controls (see Fig 7B). The maximum cross‐sectional area of myelin loss as determined by LFB staining, and confirmed in resin sections, was significantly correlated with maximum cross‐sectional area as assessed by MRI (*p* < 0.001, *r* = 0.958).

## Discussion

We have studied factors that precede the formation of an experimental demyelinating lesion that appears to be an accurate model of the MS lesion described in different studies as the “initial,” “primary,” “primordial,” “prephagocytic,” or “pattern III” demyelinating lesion. The findings illuminate why, how, and where the experimental lesion forms, and we propose that pattern III lesions in MS may form by similar mechanisms. Understanding how the experimental lesion is formed has revealed a novel therapeutic strategy to prevent the demyelination from occurring. Notably, hypoxia emerges as a decisive constituent of the factors causing pattern III demyelination in our experiments. Thus our earlier and current observations together implicate tissue hypoxia as playing a key role in 2 of the most important aspects of MS, namely the production of neurological deficits,^29^ and at least some of the demyelination.

### Why?

We have employed the endotoxin LPS to provoke an innate inflammatory reaction that is well established as initiating the release of a number of toxic mediators including superoxide and nitric oxide, and here we describe the additional, and seemingly decisive, presence of tissue hypoxia. The inflammatory reaction is followed by the appearance of a demyelinating lesion in which the demyelination is achieved by the distinctive pattern III mechanism.^4^, ^6^, ^26^ The experimental lesion is achieved by the same mechanisms as implicated in the human MS lesion,^27^, ^28^ and notably it does not seem to result from autoimmune mechanisms.

#### Potential Relevance to MS

In the LPS lesion, the demyelination results from the focal administration of a bacterial toxin, but although some have suggested that MS may arise from bacterial activity,^31^ there is no reason from our data to believe that other mechanisms that result in similar activation of the innate immune system, such as Epstein–Barr virus infection,^32^ would not also cause demyelination. If so, the precise identity of the infective agent may be less important than its ability in a particular person to induce a local environment toxic to oligodendrocytes, such as one characterized by hypoxia, nitric oxide, and superoxide. The responsible agents may alternatively not be infective, but might arise as an extreme expression of more routine events promoting hypoxia that may precipitate an energy crisis of similar magnitude and consequence. Once the lesion is initiated, acquired immune events may amplify the nascent lesion, expanding it into the “active” demyelinating lesion commonly described.

### How?

The current study implicates the expression of tissue hypoxia in association with nitric oxide and superoxide during the first 2 days after intraspinal LPS injection, and as each of these factors can impair mitochondrial function, it appears likely that their combined effect during the first 2 days is sufficient to cause an energy deficit that kills vulnerable cells such as oligodendrocytes by the third day, with the hypoxia‐like, pattern III demyelination apparent a few days later.

#### Hypoxia

Oxygen tension in the central nervous system (CNS) is normally relatively low, and within the white matter it is especially low, such that nearly anoxic values of <1mmHg have been recorded, even in normal tissue (reviewed in Ndubuizu and LaManna^33^). Therefore, cells of the CNS, particularly axons and oligodendrocytes in the white matter, habitually exist near the limit of oxidative phosphorylation. Upon the superimposition of more profound hypoxia, such as we report here, the most susceptible cells, including the oligodendrocytes, will be severely and perhaps lethally compromised, resulting in demyelination.

#### Nitric Oxide

The findings show that iNOS is prominent in the early LPS lesion,^26^, ^27^ and nitric oxide is strongly implicated in neurodegeneration^34^; many of the mechanisms thought responsible for neuronal loss will also apply to oligodendrocytes. Nitric oxide competes with oxygen for the same binding site on mitochondrial cytochrome c oxidase, raising the Michaelis constant for oxygen, so the combination of raised nitric oxide with reduced oxygen can be lethal to cells such as oligodendrocytes, even if either complication can be tolerated alone.^35^

#### Superoxide

The fluorescent labeling at the lesion following the systemic administration of DHE indicates the enhanced production of superoxide and its related cascade of reactive oxygen species. Oligodendrocytes are particularly sensitive to oxidative damage.^36^

#### Vulnerability of Oligodendrocytes to Energy Insufficiency

Selective white matter damage is a common consequence of hypoxia,^37^ ischemia,^38^ and inhibitors of mitochondrial oxidative phosphorylation,^39^ and this vulnerability can explain the pattern III demyelination observed in this study.

#### Potential Relevance to MS

Evidence of hypoxia has been reported in the MS brain, but these findings have previously been attributed to “virtual hypoxia,”^7^ arising not from a low oxygen concentration but rather from nitric oxide‐mediated mitochondrial inhibition.^34^ The mechanisms underlying virtual hypoxia are likely to play a role, but here we introduce the new finding that a state of actual hypoxia, a low oxygen concentration, precedes the demyelination.

The most obvious potential cause of hypoxia in MS is inadequate vascular perfusion, which does not necessarily imply a reduction in perfusion from normal levels, but several studies have nonetheless revealed cerebral hypoperfusion in MS (recently reviewed in D'Haeseleer et al,^22^ Juurlink,^23^ Paling et al^24^).

### Where?

A surprising feature of the LPS lesion is its location. Intraspinal injection of demyelinating agents typically results in lesions centered on the site of injection, but the demyelination resulting from LPS injection instead extends along the white matter–gray matter border and often into the base of the dorsal columns. This shift in location is noticeable and consistent, and it implies that the LPS does not cause the lesion directly, but rather that it initiates a sequence of events culminating in lesion formation at the neighboring site a week later.

The shift in lesion location from the site of injection correlates with our observations that the white matter–gray matter border at the base of the dorsal columns is inherently susceptible to hypoxia, and that even in normal animals it becomes selectively hypoxic upon simply breathing 10% oxygen. This is likely a consequence of the relative paucity of blood vessels in this region, and this will be exacerbated by the gross vascular anatomy of the spinal cord, namely that, as in humans, the base of the dorsal columns is located at a watershed between the terminal branches of 3 arterial supplies.^40^, ^41^ In support of this notion, watershed tissue is the first to lose perfusion, forming watershed infarcts, upon a global reduction in vascular supply, such as due to a reduction in blood pressure.^42^

In addition to being located at a watershed, the base of the dorsal column is also at risk because it is supplied by blood that has been partially or largely deoxygenated during its prior perfusion through the hypoxic gray matter. It seems that the depleted blood contains insufficient oxygen to maintain oligodendrocyte vitality, especially in the presence of nitric oxide and superoxide, resulting in the demyelination observed.

We propose that the coincident presence of hypoxia, nitric oxide, and superoxide results in the initiation of events culminating in oligodendrocyte loss, and ensuing primary demyelination, in the LPS lesion. Certainly the spatial distribution of these factors predicts the exact location of the demyelinated lesion that forms approximately 1 week later.

#### Potential Relevance to MS

The principles established in the LPS lesion of the rat spinal cord can be extrapolated to the human brain, where a system of nonanastomosing end‐arteries penetrates from the brain surface to supply the deeper tissues, which contain a low density of vessels.^17^, ^43^ The vascular anatomy imposes some well‐known watersheds or border zones, such as between the various cerebral arteries,^17^ along the spinal cord^44^ and the optic nerves,^45^ and particularly, involving the periventricular white matter (a distal irrigation field).^17^ It is notable that these watershed regions are prone to MS lesions (intercerebral arteries,^17^, ^18^, ^21^ spinal cord,^15^ optic nerve,^16^ and periventricular white matter^17^), although conditions that favor lesion formation can also arise from microwatersheds between smaller arteries, and in nonwatershed tissue. The tendency of lesions to form in watersheds has recently been reported by 2 independent groups, which have published heat maps revealing that approximately 90%^21^ or 86 to 100%^18^ of their patients had lesions in the watershed between the anterior and middle cerebral arteries. Interestingly, MRI reveals that blood flow in the cerebral border zone regions in healthy individuals is significantly lower than in non–border zone regions, with longer arterial transit times.^46^ Long arterial transit times substantially increase the vulnerability of arterial blood to become deoxygenated by supplying oxygen to surrounding tissue, particularly if it is hypoxic.

Veins are present at higher density in the deep white matter and they can contain such deoxygenated blood that they become sinks for oxygen,^47^ draining it from the surrounding oligodendrocytes and axons, promoting a hypoxic environment. In contrast to the earlier suspicion that “something bad leaks from veins” to cause perivenular demyelination, perhaps something good, oxygen, fails to leak from veins.

### A View of How Some MS Lesions May Originate

We suspect that a number of mechanisms may be involved in the formation of MS lesions, and that the balance of mechanisms may vary between individuals based on physiological and genetic factors, and even between lesions in the same individual. However, we suggest that one mechanism that has been insufficiently considered involves the activation of innate immune mechanisms, perhaps by a local or systemic infection, impaired perfusion, or the coincidence of physiological events that may collectively compromise the local environment or oxygen balance. It remains possible that, in MS, innate immune mechanisms are activated in response to an autoimmune attack, but the apparent paucity of T cells in the early pattern III lesion in MS, and the seeming absence of a need for autoimmunity in the laboratory model, argue against this possibility in pattern III MS lesions. The activation of innate immune mechanisms will promote an energy deficit arising, in part, from mitochondrial dysfunction induced by tissue hypoxia and the influence of nitric oxide and superoxide. Breakdown of the blood–brain barrier will likely ensue, and will augment microglial activation via exposure to plasma proteins. In this environment, an imbalance between oxygen supply and demand may overwhelm and outpace the mechanisms responsible for oxygen homeostasis, eroding the safety factor for oxygen and exposing vulnerable areas such as those located at poorly vascularized, watershed regions at the ends of long arterial trees.^41^, ^48^ Oligodendrocytes will be damaged, resulting in frank demyelination a few days later, and axons may also succumb, resulting in degeneration.^26^ The demyelination will result in exposure of normal and modified myelin antigens, which, in appropriate individuals, may incite an acquired immune reaction (see eg Traka et al^49^) that further promotes hypoxia, superoxide, and nitric oxide, inducing the familiar actively demyelinating lesion that appears in association with lymphocytes. The importance of hypoxia in causing the demyelination is emphasized by our ability to prevent demyelination by augmenting oxygen delivery during the vulnerable period.

The selective vulnerability of the white matter to hypoxia means that oligodendrocytes are more likely to suffer from hypoxia than neurons, due to their location, resulting in demyelination rather than degeneration. Axonal integrity within a focal hypoxic region may be protected by the presence of lengths of the same axons still residing in normally oxygenated tissue.

### Therapy

It is striking that the demyelination can be greatly reduced, or even prevented, by simply raising inspired oxygen at normobaric pressure for the first 2 days when the lesion is vulnerable to hypoxia. This finding suggests a key role for hypoxia in the formation of pattern III lesions. (The earlier clinical trials of hyperbaric oxygen were not designed to prevent demyelination, and the administration of oxygen was applied randomly with regard to lesion formation.) Therapeutically, it is encouraging that oxygen is easily administrated and there is substantial clinical evidence that oxygen administration is generally safe, if delivered at moderate concentration and duration. However, there is a theoretical safety concern that increasing oxygenation could promote oxidative damage, which is already implicated in MS pathology.^30^, ^50^, ^51^ Furthermore, by analogy with reperfusion injury, it is possible that the reoxygenation of hypoxic tissue might particularly promote oxidative stress. In the current experiments the oxygenation will have avoided hypoxia in the first place, and so the therapy will not have reversed ongoing hypoxia, as would be the case with oxygen therapy applied early in lesion formation in MS. Conversely, it is possible that the superoxide production observed in the inflamed tissue may be a consequence of the hypoxia itself,^52^ in which case the oxidative stress may be avoided, rather than enhanced, by oxygenation. The safety of oxygenation, including the reversal of ongoing hypoxia, are current topics of investigation in our laboratory, and we caution against the speculative use of oxygen as an acute therapy until the safety is understood. Aside from the administration of oxygen gas there are other potential avenues that can be considered, and the positive outcome of therapy with erythropoietin in optic neuritis^53^ encourages a view that such avenues may be effective.

## Author Contributions

R.A.D. and A.L.D. designed the study, and were principally responsible for conducting all the experiments, analyzing the data, and writing the manuscript. M.T. conducted the ex vivo MRI experiments and aided with manuscript preparation. X.G. supervised the MRI experiments and aided with manuscript preparation. M.K. and F.L. provided preliminary observations. K.J.S. supervised the project and wrote the manuscript. All authors approved the final manuscript before submission. R.A.D. and A.L.D. are joint first authors.

## Potential Conflicts of Interest

K.J.S. has received research funding for unrelated work from Biogen Idec and Merck Serono.

## Supporting information

Additional supporting information can be found in the online version of this article.

Supporting Information Table 1.

## References

[1] Prineas JW , Parratt JD . Oligodendrocytes and the early multiple sclerosis lesion. Ann Neurol 2012;72:18–31. 22829266 10.1002/ana.23634

[2] Henderson AP , Barnett MH , Parratt JD , et al. Multiple sclerosis: distribution of inflammatory cells in newly forming lesions. Ann Neurol 2009;66:739–753. 20035511 10.1002/ana.21800

[3] Gay FW , Drye TJ , Dick GW , et al. The application of multifactorial cluster analysis in the staging of plaques in early multiple sclerosis. Identification and characterization of the primary demyelinating lesion. Brain 1997;120:1461–1483. 9278635 10.1093/brain/120.8.1461

[4] Lucchinetti C , Bruck W , Parisi J , et al. Heterogeneity of multiple sclerosis lesions: implications for the pathogenesis of demyelination. Ann Neurol 2000;47:707–717. 10852536 10.1002/1531-8249(200006)47:6<707::aid-ana3>3.0.co;2-q

[5] Lassmann H , van Horssen J , Mahad D . Progressive multiple sclerosis: pathology and pathogenesis. Nat Rev Neurol 2012;8:647–656. 23007702 10.1038/nrneurol.2012.168

[6] Aboul‐Enein F , Lassmann H . Mitochondrial damage and histotoxic hypoxia: a pathway of tissue injury in inflammatory brain disease? Acta Neuropathol 2005;109:49–55. 15645261 10.1007/s00401-004-0954-8

[7] Trapp BD , Stys PK . Virtual hypoxia and chronic necrosis of demyelinated axons in multiple sclerosis. Lancet Neurol 2009;8:280–291. 19233038 10.1016/S1474-4422(09)70043-2

[8] Lassmann H , Reindl M , Rauschka H , et al. A new paraclinical CSF marker for hypoxia‐like tissue damage in multiple sclerosis lesions. Brain 2003;126:1347–1357. 12764056 10.1093/brain/awg127

[9] McMahon JM , McQuaid S , Reynolds R , et al. Increased expression of ER stress‐ and hypoxia‐associated molecules in grey matter lesions in multiple sclerosis. Mult Scler 2012;18:1437–1447. 22354737 10.1177/1352458512438455

[10] Aboul‐Enein F , Rauschka H , Kornek B , et al. Preferential loss of myelin‐associated glycoprotein reflects hypoxia‐like white matter damage in stroke and inflammatory brain diseases. J Neuropathol Exp Neurol 2003;62:25–33. 12528815 10.1093/jnen/62.1.25

[11] Lee Y , Morrison BM , Li Y , et al. Oligodendroglia metabolically support axons and contribute to neurodegeneration. Nature 2012;487:443–448. 22801498 10.1038/nature11314 PMC3408792

[12] Okeda R , Song SY , Funta N , et al. An experimental study of the pathogenesis of Grinker's myelinopathy in carbon monoxide intoxication. Acta Neuropathol 1983;59:200–206. 6845982 10.1007/BF00703204

[13] Campbell GR , Mahad DJ . Mitochondrial changes associated with demyelination: consequences for axonal integrity. Mitochondrion 2012;12:173–179. 21406249 10.1016/j.mito.2011.03.007

[14] Zimmerman HM , Netsky MG . The pathology of multiple sclerosis. Res Publ Assoc Res Nerv Ment Dis 1950;28:271–312. 15413018

[15] DeLuca GC , Williams K , Evangelou N , et al. The contribution of demyelination to axonal loss in multiple sclerosis. Brain 2006;129:1507–1516. 16597651 10.1093/brain/awl074

[16] Toosy AT , Mason DF , Miller DH . Optic neuritis. Lancet Neurol 2014;13:83–99. 24331795 10.1016/S1474-4422(13)70259-X

[17] Brownell B , Hughes JT . The distribution of plaques in the cerebrum in multiple sclerosis. J Neurol Neurosurg Psychiatry 1962;25:315–320. 14016083 10.1136/jnnp.25.4.315 PMC495470

[18] Haider L , Zrzavy T , Hametner S , et al. The topography of demyelination and neurodegeneration in the multiple sclerosis brain. Brain 2016;awv398. 10.1093/brain/awv398 PMC4766379 26912645

[19] Lazorthes G . Vascularisation et circulation cérébrales. Paris, France: Masson, 1961.

[20] Petersen ET , Mouridsen K , Golay X . The QUASAR reproducibility study, part II: results from a multi‐center arterial spin labeling test‐retest study. Neuroimage 2010;49:104–113. 19660557 10.1016/j.neuroimage.2009.07.068 PMC2768325

[21] Holland CM , Charil A , Csapo I , et al. The relationship between normal cerebral perfusion patterns and white matter lesion distribution in 1,249 patients with multiple sclerosis. J Neuroimaging 2012;22:129–136. 21447022 10.1111/j.1552-6569.2011.00585.x

[22] D'Haeseleer M , Hostenbach S , Peeters I , et al. Cerebral hypoperfusion: a new pathophysiologic concept in multiple sclerosis? J Cereb Blood Flow Metab 2015;35:1406–1410. 26104292 10.1038/jcbfm.2015.131 PMC4640326

[23] Juurlink BH . The evidence for hypoperfusion as a factor in multiple sclerosis lesion development. Mult Scler Int 2013;2013:598093. 23691321 10.1155/2013/598093 PMC3649777

[24] Paling D , Thade PE , Tozer DJ , et al. Cerebral arterial bolus arrival time is prolonged in multiple sclerosis and associated with disability. J Cereb Blood Flow Metab 2014;34:34–42. 24045400 10.1038/jcbfm.2013.161 PMC3887342

[25] Lumsden CE . The neuropathology of multiple sclerosis In: Vinken PJ, Bruyn GW, eds. Handbook of clinical neurology. Amsterdam, the Netherlands: Elsevier, 1970:217–309.

[26] Felts PA , Woolston AM , Fernando HB , et al. Inflammation and primary demyelination induced by the intraspinal injection of lipopolysaccharide. Brain 2005;128:1649–1666. 15872019 10.1093/brain/awh516 PMC7109778

[27] Marik C , Felts PA , Bauer J , et al. Lesion genesis in a subset of patients with multiple sclerosis: a role for innate immunity? Brain 2007;130:2800–2815. 17956913 10.1093/brain/awm236 PMC2981817

[28] Sharma R , Fischer MT , Bauer J , et al. Inflammation induced by innate immunity in the central nervous system leads to primary astrocyte dysfunction followed by demyelination. Acta Neuropathol 2010;120:223–236. 20532539 10.1007/s00401-010-0704-z PMC2892605

[29] Davies AL , Desai RA , Bloomfield PS , et al. Neurological deficits caused by tissue hypoxia in neuroinflammatory disease. Ann Neurol 2013;74:815–825. 24038279 10.1002/ana.24006

[30] Schuh C , Wimmer I , Hametner S , et al. Oxidative tissue injury in multiple sclerosis is only partly reflected in experimental disease models. Acta Neuropathol 2014;128:247–266. 24622774 10.1007/s00401-014-1263-5 PMC4102830

[31] Gay F . Staphylococcal immune complexes and myelinolytic toxin in early acute multiple sclerosis lesions—an immunohistological study supported by multifactorial cluster analysis and antigen‐imprint isoelectric focusing. Mult Scler Relat Disord 2013;2:213–232. 25877728 10.1016/j.msard.2013.01.002

[32] Pender MP . The essential role of Epstein‐Barr virus in the pathogenesis of multiple sclerosis. Neuroscientist 2011;17:351–367. 21075971 10.1177/1073858410381531 PMC3764840

[33] Ndubuizu O , LaManna JC . Brain tissue oxygen concentration measurements. Antioxid Redox Signal 2007;9:1207–1219. 17536959 10.1089/ars.2007.1634

[34] Brown GC . Nitric oxide and neuronal death. Nitric Oxide 2010;23:153–165. 20547235 10.1016/j.niox.2010.06.001

[35] Mander P , Borutaite V , Moncada S , et al. Nitric oxide from inflammatory‐activated glia synergizes with hypoxia to induce neuronal death. J Neurosci Res 2005;79:208–215. 15558752 10.1002/jnr.20285

[36] Juurlink BH . Response of glial cells to ischemia: roles of reactive oxygen species and glutathione. Neurosci Biobeh Rev 1997;21:151–166. 10.1016/s0149-7634(96)00005-x 9062938

[37] Lyons SA , Kettenmann H . Oligodendrocytes and microglia are selectively vulnerable to combined hypoxia and hypoglycemia injury in vitro. J Cereb Blood Flow Metab 1998;18:521–530. 9591844 10.1097/00004647-199805000-00007

[38] Petito CK , Olarte JP , Roberts B , et al. Selective glial vulnerability following transient global ischemia in rat brain. J Neuropathol Exp Neurol 1998;57:231–238. 9600215 10.1097/00005072-199803000-00004

[39] Tsutsui S , Stys PK . Metabolic injury to axons and myelin. Exp Neurol 2013;246:26–34. 22569104 10.1016/j.expneurol.2012.04.016

[40] Koyanagi I , Tator CH , Lea PJ . Three‐dimensional analysis of the vascular system in the rat spinal cord with scanning electron microscopy of vascular corrosion casts. Part 2: Acute spinal cord injury. Neurosurgery 1993;33:285–291. 8367052

[41] Tveten L . Spinal cord vascularity. IV. The spinal cord arteries in the rat. Acta Radiol Diagn (Stockh) 1976;17:385–398. 970203 10.1177/028418517601700401

[42] Momjian‐Mayor I , Baron JC . The pathophysiology of watershed infarction in internal carotid artery disease: review of cerebral perfusion studies. Stroke 2005;36:567–577. 15692123 10.1161/01.STR.0000155727.82242.e1

[43] Moody DM , Bell MA , Challa VR . Features of the cerebral vascular pattern that predict vulnerability to perfusion or oxygenation deficiency: an anatomic study. AJNR Am J Neuroradiol 1990;11:431–439. 2112304 PMC8367475

[44] Shamji MF , Maziak DE , Shamji FM , et al. Circulation of the spinal cord: an important consideration for thoracic surgeons. Ann Thorac Surg 2003;76:315–321. 12842576 10.1016/s0003-4975(03)00139-5

[45] Hayreh SS . Blood supply of the optic nerve. Ischemic Optic Neuropathies 2011;35–78.

[46] Hendrikse J , Petersen ET , van Laar PJ , et al. Cerebral border zones between distal end branches of intracranial arteries: MR imaging. Radiology 2008;246:572–580. 18055872 10.1148/radiol.2461062100

[47] Ivanov KP , Sokolova IB , Vovenko EP . Oxygen transport in the rat brain cortex at normobaric hyperoxia. Eur J Appl Physiol Occup Physiol 1999;80:582–587. 10541925 10.1007/s004210050637

[48] Martirosyan NL , Feuerstein JS , Theodore N , et al. Blood supply and vascular reactivity of the spinal cord under normal and pathological conditions. J Neurosurg Spine 2011;15:238–251. 21663407 10.3171/2011.4.SPINE10543

[49] Traka M , Podojil JR , McCarthy DP , et al. Oligodendrocyte death results in immune‐mediated CNS demyelination. Nat Neurosci 2016;19:65–74. 26656646 10.1038/nn.4193 PMC4837900

[50] Fischer MT , Sharma R , Lim JL , et al. NADPH oxidase expression in active multiple sclerosis lesions in relation to oxidative tissue damage and mitochondrial injury. Brain 2012;135:886–899. 22366799 10.1093/brain/aws012 PMC3286337

[51] Smith KJ , Lassmann H . The role of nitric oxide in multiple sclerosis. Lancet Neurol 2002;1:232–241. 12849456 10.1016/s1474-4422(02)00102-3

[52] Murphy MP . How mitochondria produce reactive oxygen species. Biochem J 2009;417:1–13. 19061483 10.1042/BJ20081386 PMC2605959

[53] Suhs KW , Hein K , Sattler MB , et al. A randomized, double‐blind, phase 2 study of erythropoietin in optic neuritis. Ann Neurol 2012;72:199–210. 22926853 10.1002/ana.23573

