# Supplementary material for: Cause and prevention of demyelination in a model multiple sclerosis lesion
Source: Ann Neurol. 2016 Feb 22;79(4):591–604. doi: 10.1002/ana.24607 (PMC4949637; doi:10.1002/ana.24607)
Supplement: Supplementary file 1 — Supporting Information Table 1. [file ANA-79-591-s001.docx]

**Table 1. Immunohistochemsitry, antibodies, and protocols**

| **Antibody** | **Target** | **Isotype** | **Pre-Treatment** | **Blocking Buffer** | **Dilution** | **Source** |
| --- | --- | --- | --- | --- | --- | --- |
| Pimonidazole | Pimonidazole adducts | Mouse IgG_1_ | 1mg/ml NaBH_4_ | 0.25% casein (VWR) in 0.1% PBS-triton | 1:500 | HPI Inc  HP1-1000kit |
| APC (CC-1) | Adenomatous Polyposis Coli  (oligodendrocytes) | Mouse IgG_2b_ | None | 5% horse serum in 0.01% PBS-triton | 1:200 | Abcam  ab16794 |
| Nitrotyrosine | Nitrotyrosine | Mouse IgG_2b_ | Citrate buffer, pH6. (DAKO) | 5% horse serum in 0.01% PBS-triton | 1:200 | Millipore  MAB5404 |
| Pimonidazole | Pimonidazole adducts | Rabbit IgG | 1mg/ml NaBH_4_ | 5% goat serum (Vector Labs) in 0.01% PBS-triton | 1:200 | HPI Inc  HP3-1000kit |
| iNOS | Inducible nitric oxide synthase | Rabbit IgG | None | 5% goat serum in 0.01% PBS-triton | 1:200 | BD Transductions Ltd  610333 |
| GFAP | Astrocytes | Rabbit IgG | 1mg/ml NaBH_4_ | 5% goat serum in 0.01% PBS-triton | 1:500 | Dako  ZO334 |
| HIF-1α | Hypoxia inducible factor-1 α | Rabbit IgG | 1mg/ml NaBH_4_ | 5% goat serum in 0.01% PBS-triton | 1:500 | Millipore  07-628 |
| IBA | Macrophages/microglia | Rabbit IgG | 1mg/ml NaBH_4_ | 5% goat serum in 0.01% PBS-triton | 1:200 | Wako Chemicals  019-19741 |
| CA2 | Carbonic anhydrase 2  (oligodendrocytes) | Rabbit IgG | None | 5% goat serum in 0.01% PBS-triton | 1:200 | Kind gift from Dr. N. Gregson |
